# Relating normal human cardiac development to the anatomical findings in the congenitally malformed heart

**DOI:** 10.1002/ca.24240

**Published:** 2024-11-13

**Authors:** Jill P. J. M. Hikspoors, Wouter H. Lamers, Janet Kerwin, Zihan Hu, Deborah J. Henderson, Robert H. Anderson

**Affiliations:** ^1^ Department of Anatomy & Embryology Maastricht University Maastricht The Netherlands; ^2^ Tytgat Institute for Liver and Intestinal Research Academic Medical Center Amsterdam The Netherlands; ^3^ Biosciences Institute Newcastle University Newcastle upon Tyne UK

**Keywords:** cardiac compartment, cardiac loop, cardiac septation, developmental timing, embryonic ventricle, growth, staging

## Abstract

A proper appreciation of cardiac development can now provide the necessary background to understand the anatomical findings in the congenitally malformed heart. We recently presented an account of human cardiac development based on reconstructions of histological datasets from human embryos aged between 3.5 and 8 weeks subsequent to conception. In this review, we summarize the changes observed relative to the findings when the heart is congenitally malformed. Beginning at the stage when it is first possible to recognize the primary heart tube, we describe the looping of its ventricular component, which occurs in the 5th week. We proceed with discussion of the formation of the atrial and ventricular chambers in the 6th week. The phases are successive, albeit partially overlapping. Separation of the circulations at the venous pole is completed at stage 17, equivalent to almost 6 weeks of development. During stages representing the 7th week of development, we concentrate on the remodeling of the outflow tract. This involves initially septation, but then separation of the developing circulations. The changes involve incorporation of the proximal outflow tract into the ventricles, with formation of the arterial roots in its middle part, and addition of a distal non‐myocardial component to produce the intrapericardial arterial trunks. We pay particular attention to the changes occurring during remodeling of the interventricular foramen. We show that an understanding of this process provides the basis for understanding the functionally univentricular heart, as well as the arrangement found in double outlet right ventricle.

## 1 INTRODUCTION

Our understanding of cardiac development, over recent decades, has increased significantly due to studies of mammalian embryos, in particular mice. Knowledge of human development has been more limited, although this has long been recognized as providing the basis for understanding the many and varied congenital cardiac malformations (Abbott, 1934). The required information has usually depended on examination of serial sections stained with classical dyes, although more recently studies have permitted the identification of the location of key genes (Sizarov et al., 2010; Wessels et al., 1990). It is difficult, nonetheless, to appreciate the significant changes that take place with the passage of time simply by assessing the sections themselves. We have now had the opportunity to reconstruct the hearts of 12 embryos available within the Digitally Reproduced Embryonic Morphology, or DREM, project (Gasser et al., 2014). The embryos, which cover the period between 3.5 and 8 weeks of development subsequent to fertilization, are part of the Carnegie collection. They were staged a long time ago by experienced embryologists, who did not have cardiac development in mind when assigning a stage to an embryo. This permitted us to assign critical events in cardiac development to specific Carnegie stages. The files are now available as freely accessible and fully interactive datasets, which can be inspected on any computer with a PDF reader installed (Hikspoors et al., 2022); they are also available within the HDBR Atlas (https://hdbratlas.org/). The reconstructed models can be deconstructed, rotated, and magnified. Throughout the series, each structure retains the same color code, while a scale cube permits an estimation of its size. We also now have access to the material available in the Human Developmental Biology Resource (see Anderson, Kerwin, et al., 2024), along with some of the embryonic and fetal hearts which were stained to show the locations of key proteins (Sizarov et al., 2010; Wessels et al., 1990). We have used all this material to prepare this review, in which we show how the changes occurring during normal development provide the basis for understanding the morphology of congenital cardiac malformations, in particular the basis of sequential segmental analysis (Anderson et al., 1984). In our initial account (Hikspoors et al., 2022), we described the developmental appearance, and subsequent remodeling, of the structures as seen during each of the developmental stages. For this review, we have taken a different stance. We begin our accounting by describing the arrangement when there is but a single heart tube. Having described the looping of its ventricular component, we discuss the formation of the atrial and ventricular chambers, emphasizing the role of remodeling of the interventricular communication in formation of the ventricles. We then explain how the various compartments are separated to produce the discrete pulmonary and systemic circulations, introducing at this stage the significant remodeling required in the outlet component. We discuss how all this information now provides a rational explanation for many, if not most, of the varied lesions found when the heart is congenitally malformed.

## 2 FORMATION OF THE SINGLE HEART TUBE

After around 23 days of development subsequent to conception, which is equivalent to Carnegie stage 8, the embryo is no more than a dorsally convex disc, sandwiched between the yolk sac ventrally, and the amniotic sac dorsally. It is at this stage, nonetheless, that the onset of gastrulation heralds the establishment of the basic body plan. The bilateral heart fields, also known as the cardiogenic plates, become defined in the early phase of gastrulation, forming craniolaterally in the mesoderm (Tam et al., 1997). They give rise to the precursors of most of the cardiac structures, albeit that not all are formed at the same time. During the transition from Carnegie stages 8–9, cells positive for the transcription factor Mesp1 produce the first heart field (Lescroart et al., 2014). This field gives rise mostly to the cells that will form the embryonic left ventricle. Toward the end of stage 9, the Mesp1‐positive cells produce the second heart field, which is characterized by the expression of the transcription factor *Isl1* (Cai et al., 2003). This second field gives rise to virtually all cardiomyocytes of the outflow tract and the majority of cardiomyocytes found in the right ventricle. It also provides many of the atrial cardiomyocytes, and a part of the atrioventricular canal (Meilhac et al., 2004). The systemic venous sinus, which receives the venous tributaries, is the last part of the developing heart to appear, although the pulmonary vein does not canalize until the sinus is incorporated in the developing right atrium. The venous sinus does not express the early cardiogenic transcription factor Nkx2‐5 but does express Tbx18 (Christoffels et al., 2006).

**FIGURE 1:**
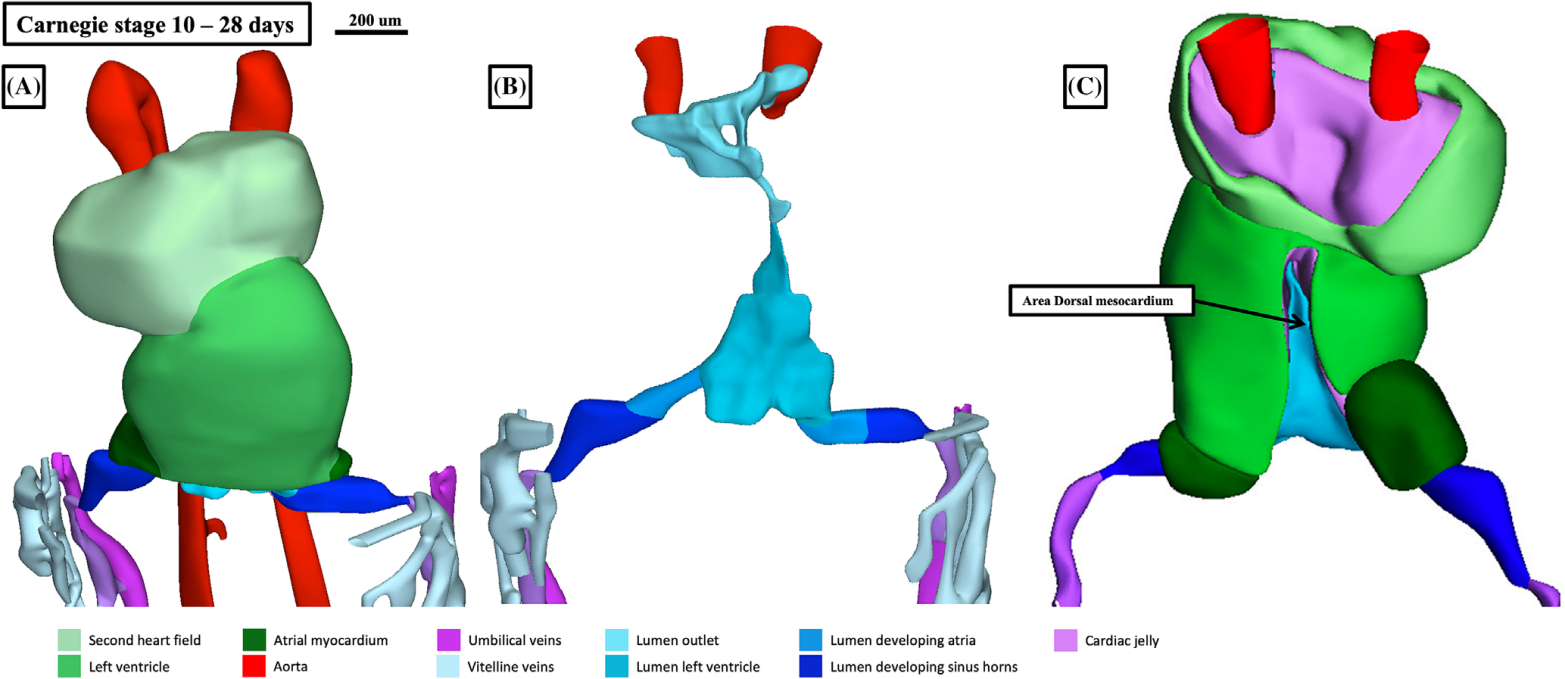
The images are created using the interactive pdf files included in the pictorial essay prepared by Hikspoors et al. (2022). The colors are retained throughout the subsequent figures prepared using the files, and a color‐coding legend is enclosed to each Figure. Panel (A) shows the myocardial walls of the straight heart tube found at Carnegie stage 10 as seen from the front. Panel (B) shows the lumens of the solitary cavity present at this stage. Panel (C) shows the reconstruction as viewed from behind, revealing the extent of the dorsal mesocardium.

It is during the tenth Carnegie stage, at around 28 days of development, that it becomes possible to recognize a developing organ enclosed within a pericardial cavity, with a single endothelial channel connecting bilateral venous and arterial pathways (Figure 1A). By this time, the initial part of the tube, which will become the left ventricle, is flanked by inflow and outflow components. These new parts have arisen from the venous and arterial parts of the second heart field (Figure 1B). As yet, however, these flanking components cannot be recognized morphologically as atrial or right ventricular structures. The endothelial tube itself is enveloped by myocardial walls, which surround the tube in the fashion of a cloak that is open dorsally (Figure 1C). The area lacking myocardium is the dorsal mesocardium, where the walls of the developing heart are in continuity with the pharyngeal mesenchyme. It extends cranially to the level of the narrow part of the hourglass‐shaped endocardial lumen (Figure 1B). This site, subsequent to looping of the heart tube, will mark the transition from the components derived from the first as opposed to the second heart field (Miquerol & Kelly, 2013).

## 3 FORMATION OF THE VENTRICULAR LOOP

**FIGURE 2:**
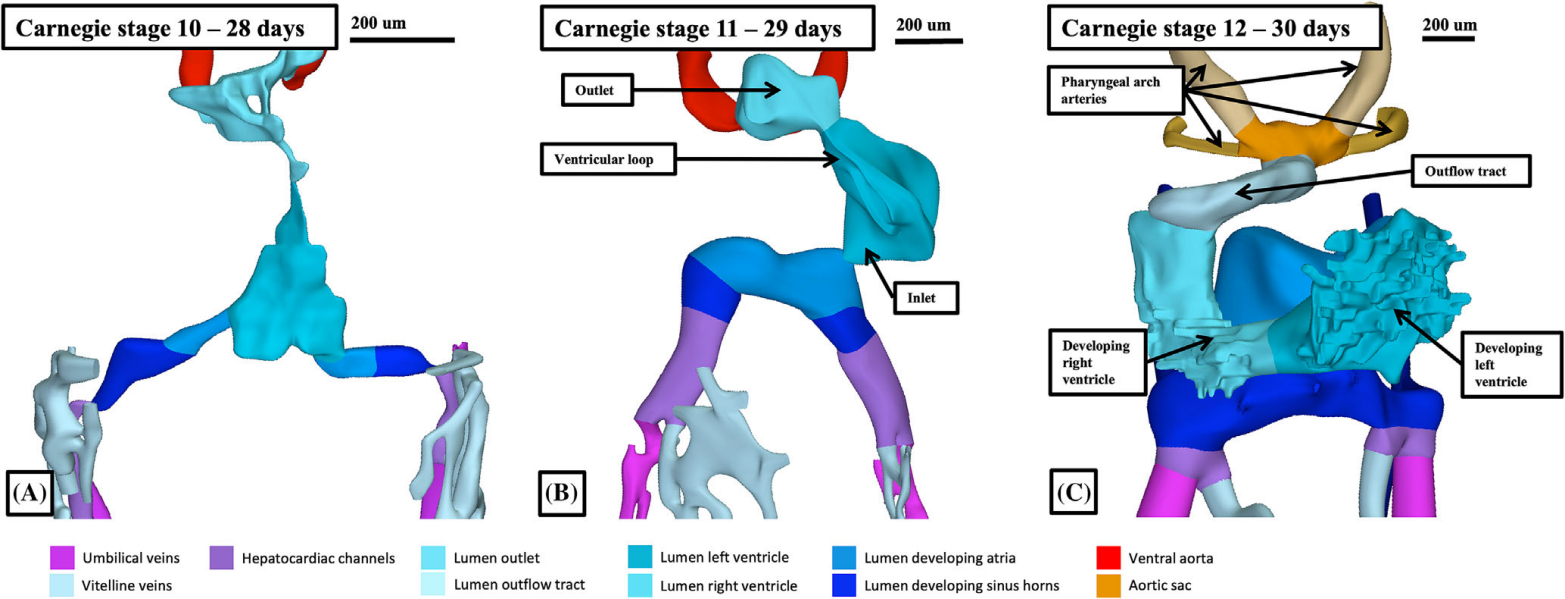
The reconstructions show the changes in the lumen of the heart tube during the process of ventricular looping. Each reconstruction is viewed from the front.

As shown in Figure 1, at Carnegie stage 10 the heart tube is straight. The developing left ventricle, at this stage, is flanked cranially by the developing right ventricle and outflow tract. Caudally, it is bordered by the venous inflows, which initially enter the tube in bilaterally symmetrical fashion (Figure 2A). During stage 11, the ventricular part of the tube, losing more of its connection through the mesocardium to the pharyngeal mesenchyme, undergoes ventral expansion. This is the key process known as looping (Figure 2B). The changes reflect a higher rate of proliferation and myocardial differentiation of the epithelial layer of the second heart field in the dorsal mesocardium, along with subsequent addition of material from the second heart field at both the venous and arterial poles (de Boer et al., 2012).

**FIGURE 3:**
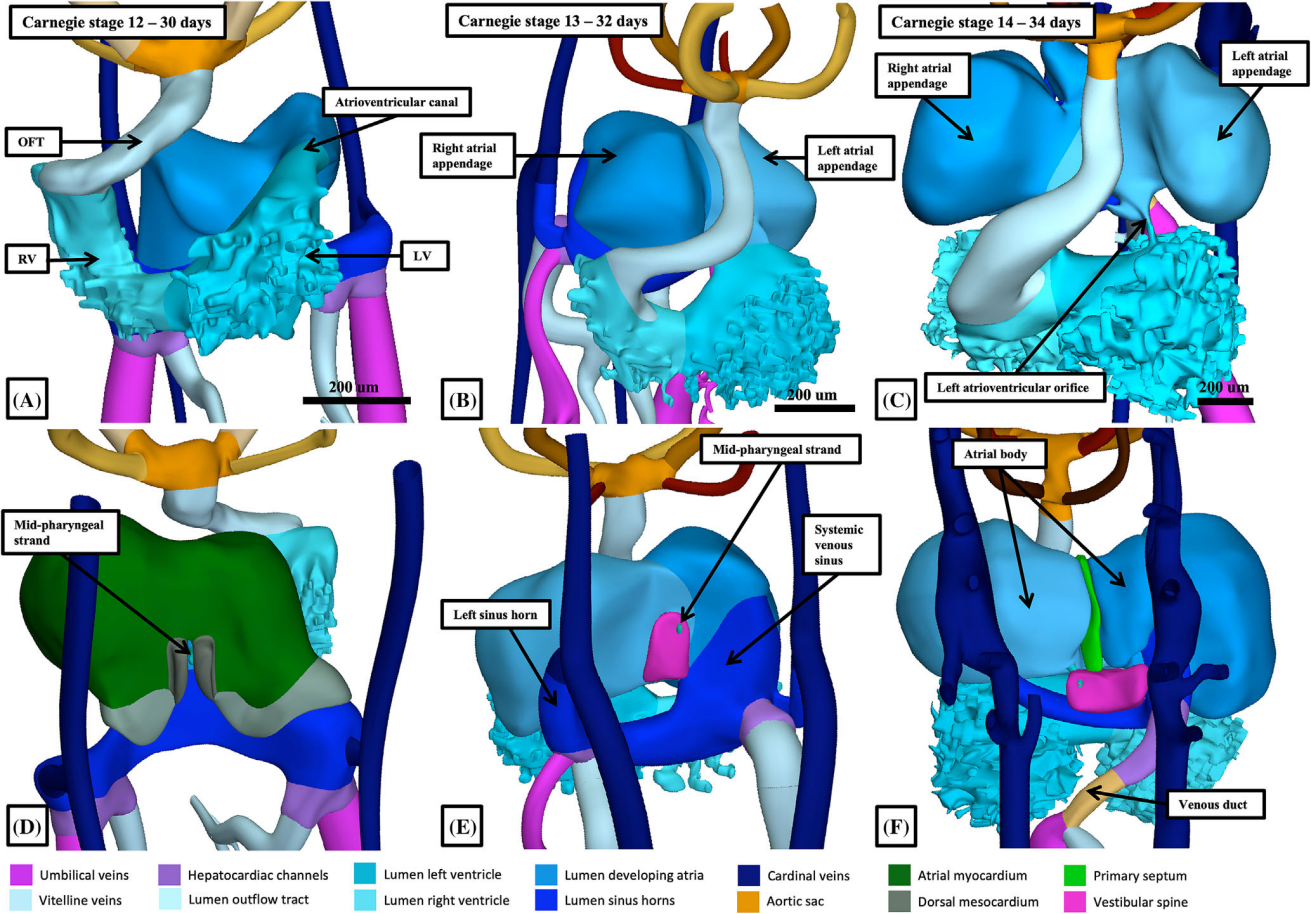
The reconstructions show the remodeling of the systemic venous tributaries taking place during Carnegie stages 12 through 14, which occupy the larger part of the 5th week of development. Panels (A)–(C) are shown from the front, with panels (D) through (E) being the same reconstructions, but shown from behind.

**FIGURE 4:**
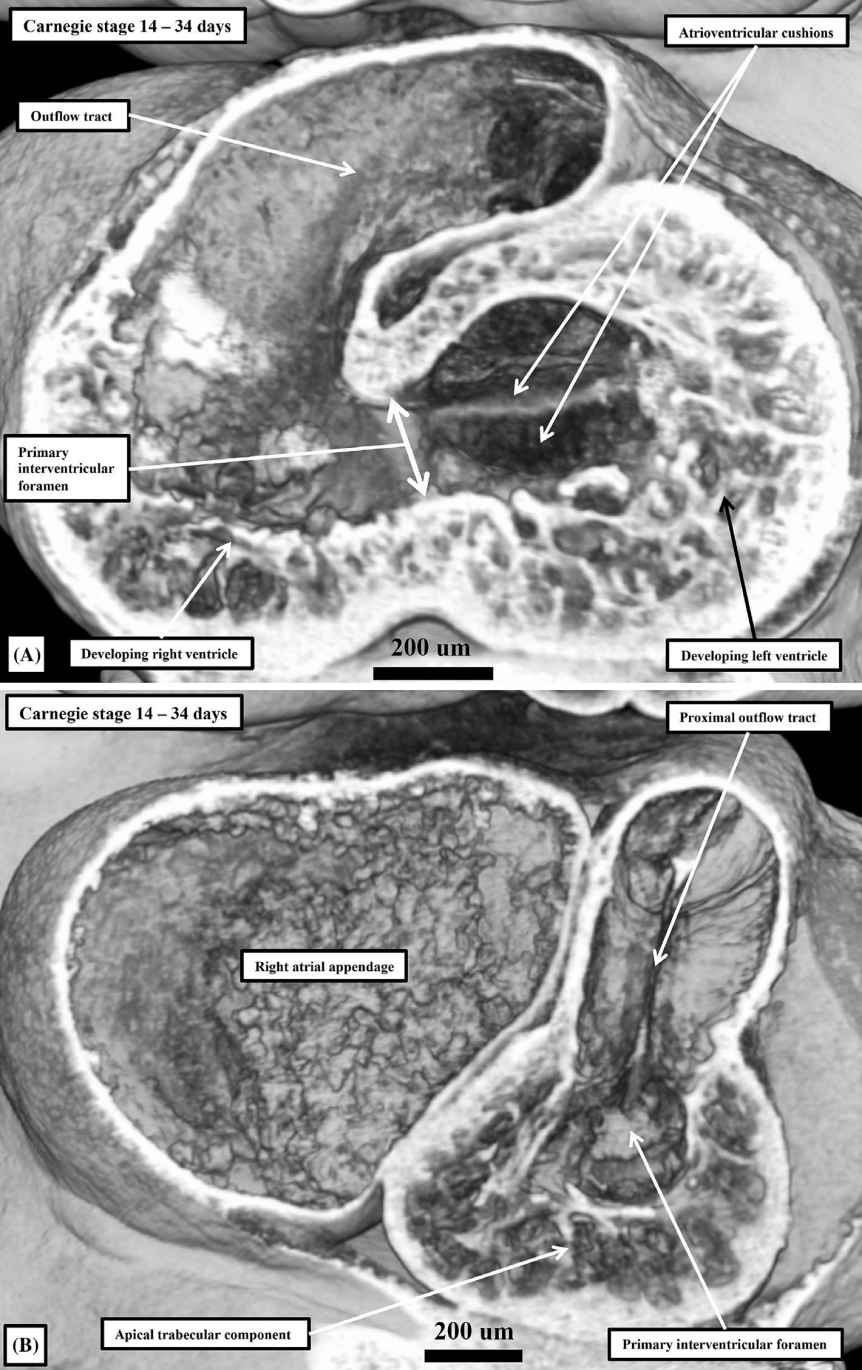
The images are prepared by taking sections through the same dataset of a human embryo at Carnegie stage 14 prepared using high‐resolution episcopic microscopy. Panel (A) was made by removing the apical part of the ventricular loop, and imaging the remaining basal components from beneath. Panel (B) is a section replicating the oblique subcostal cut obtained during echocardiographic studies. The image shown in panel (B) is reminiscent of the arrangement seen in classical tricuspid atresia when, as with the embryo at this stage, the developing right ventricle lacks its inlet component. At this stage during normal development, however, the right ventricle supports the entirety of the outlet component.

By Carnegie stage 12, which represents around 30 days of development, trabeculations can be recognized within the part of the cavity that has ballooned from the inlet part of the loop. This area will become the apical component of the developing left ventricle. Trabeculations that will eventually be found within the primordium of the apical part of the developing right ventricle have also, by now, begun to appear within the part of the cavity that balloons from the outlet part of the ventricular loop (Figure 2C). At this initial stage of looping, the component that will become the atrioventricular canal opens exclusively into the cavity of the developing left ventricle, while the outlet component of the primary heart tube is supported exclusively by the developing right ventricle (Figures 3A and 4). The outflow tract extends at this stage to the margins of the pericardial cavity, where its cavity is continuous with the primordium of the aortic sac (Figure 3B). The primordium, at this stage, gives rise, in symmetrical fashion, to the arteries of the first two pharyngeal arches. Throughout these stages, the outflow tract has an obvious bend, permitting description of proximal and distal parts. Not until stage 15, subsequent to the ongoing addition of material from the second heart field, does it become possible to recognize non‐myocardial components of the outflow tract. As we will describe subsequently, with the addition distally of the non‐myocardial part, the outflow tract itself becomes tripartite. Its proximal and middle parts then still have myocardial walls, with the non‐myocardial part forming the distal component.

## 4 THE FORMATION OF THE ATRIAL AND VENTRICULAR COMPONENTS

When first formed, at the straight heart tube stage, the developing organ contains relatively little of the eventual cardiac segments (Figures 1 and 2A). It is the continued localized increase in myocyte proliferation in the outer curvature of the ventricular loop, along with proliferation in the developing inflow component of the tube that underscores the formation of the definitive cardiac components. As we have already shown, this process, well described as “ballooning,” produces the apical components of the developing ventricles. It is similar ballooning from the atrial component of the tube that produces the atrial appendages (Figure 3C). The ventricular apical components, along with the atrial appendages, have walls formed of chamber myocardium. These myocardial components can be distinguished by their staining characteristics from the primary myocardium of the initial heart tube (Christoffels et al., 2000; Sizarov et al., 2010; Wessels et al., 1990). At Carnegie stage 11, when the first sign of ballooning can be seen at the inlet of the ventricular loop, it was not possible to recognize an atrial chamber as such. At Carnegie stage 12, furthermore, when the atrial component of the tube has become recognizable, the venous tributaries continue to return the blood from the vitelline and umbilical circulations in symmetrical fashion (Figure 3A). It is during this stage, after around 30 days have elapsed subsequent to conception, that the cardinal veins also join the venous inflows, which can then be recognized as the right and left sinus horns (Figure 2C). The atrial component of the heart tube itself, however, opens only to the left‐sided inlet of the ventricular loop as the atrioventricular canal (Figure 3A). The atrial component, nonetheless, is connected in the midline to the pharyngeal mesenchyme through the dorsal mesocardium (Figure 3A,D).

**FIGURE 5:**
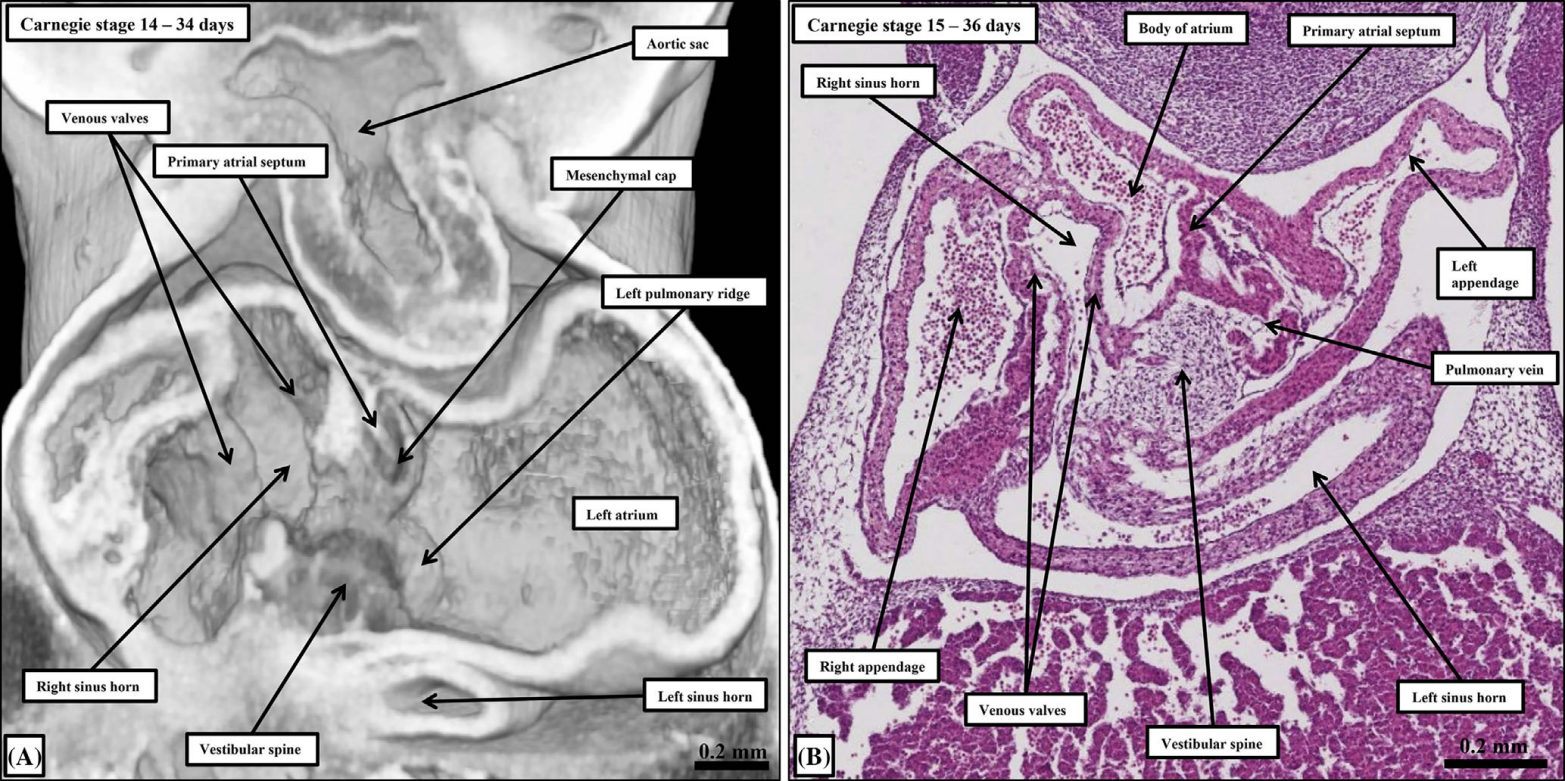
The images show the arrangement of the developing right ventricle subsequent to the incorporation of the systemic venous sinus at Carnegie stage 14. Panel (A) is a frontal section taken from an episcopic dataset, while panel (B) is a comparable histological section rotated so as to cut along the left sinus horn. Panel (B) shows how the left sinus horn has its own walls, and is located inferior to the developing walls of the left atrium.

**FIGURE 6:**
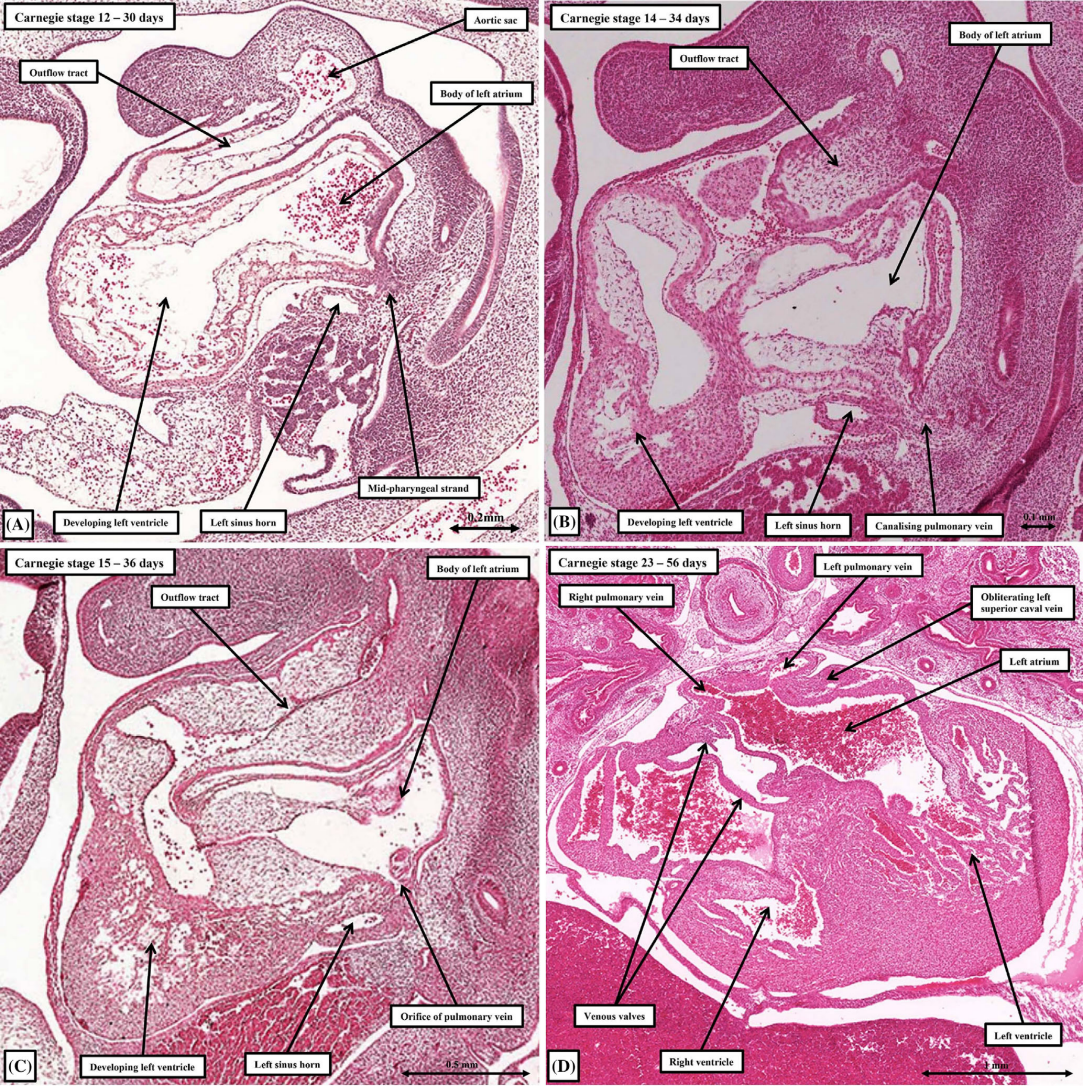
The histological sections show the stages of formation of the pulmonary venous component of the left atrium. Panels (A)–(C) are sagittal sections, whereas panel (D) is a transverse section. The transverse section shows the beginning of the superior interatrial fold.

During the transition from Carnegie stage 12 to stage 13, the realignment of the outflow tract relative to the developing atrial component of the tube has permitted the ballooning of the atrial appendages, which are made up of chamber myocardium. As they form, the right and left appendages balloon to each side of the outflow tract (Figure 3B,C). By this time, there has been a major marked change in the connections of the sinus horns to the primary atrial cavity. The walls of the left sinus horn, rotating around the fulcrum provided by the dorsal mesocardium, become positioned caudally relative to the cavity of the developing left atrium. The cavity of the left horn, however, along with that of the right horn, now opens exclusively into the right side of the developing atrial component of the initial heart tube (Figure 3E). The boundaries of this newly‐formed systemic venous sinus, which contains the openings of both sinus horns, are now marked by the right and left venous valves (Figure 5). The pulmonary vein can also be recognized subsequent to the formation of the venous valves in the developing right atrium. It canalizes from a strand located within the middle of the pharyngeal mesenchyme at the level of the dorsal mesocardium (Figure 6A). Having canalized, it initially opens within the floor of the left side of the cavity of the primary atrium, entering between the margins of the dorsal mesocardium (Figure 6B,C). It is not until almost the end of the embryonic period, however, that the pulmonary veins from the two lungs open more cranially as right‐sided and left‐sided entities (Webb et al., 2001). As we will emphasize, only at this later stage does it become possible to recognize the superior interatrial groove (Figure 6D), a structure frequently mistaken for the second atrial septum (Jensen et al., 2017).

It is the ballooning of the ventricular apical components, along with the atrial appendages, that makes it possible to recognize the definitive features of the ventricular and atrial chambers. These reflect the arrangement of the chamber, rather than the primary, myocardium. At the ventricular level, the difference between morphologically rightness and leftness depends on the coarseness of the trabeculations within the apical components, with this change not occurring until the fetal period. At the atrial level, it is the extent of the pectinate muscles, which can be seen forming within the appendages subsequent to Carnegie stage 16, relative to the developing atrioventricular junctions which provides the distinction between the morphologically left and right sides (Uemura et al., 1995). With the incorporation of the systemic venous tributaries to the right side of the primary atrial component of the initial heart tube, the majority of the primary myocardium has been incorporated into the atrial chambers as the body of the developing left atrium. The pulmonary vein initially contributes very little to the left side. It is subsequent to the growth of the primary atrial septum, to be discussed later, that it becomes possible to recognize the body of the right atrium. Again derived from the primary tube, the body of the right atrium fills the space between the left venous valve and the developing septum (Figure 5). The atrioventricular canal myocardium is also eventually incorporated into the atrial chambers. This myocardium forms the atrial vestibules. Even at these early stages, therefore, it is also possible to recognize all the components of the developing atrial and ventricular chambers. They are not, however, yet in their definitive positions. This is particularly the case for the ventricles. Initially, only the developing left ventricle possesses an inlet, while the entirety of the outlet component is supported exclusively above the developing right ventricle (Figure 4A). Already at this stage, nonetheless, it is possible to recognize the apical component of the right ventricle (Figure 4B). As we showed, this part ballooned from the outlet of the initial ventricular loop (Figure 2C). The arrangements at this stage provide the basis for functionally univentricular hearts with dominant left ventricles. The developing right ventricle, lacking its inlet component, is already comparable to the incomplete right ventricle found in the setting of tricuspid atresia (Figure 4B), particularly when compared with the variants with concordant ventriculo‐arterial connections. As in the developing heart, the essence of the commonest variant of tricuspid atresia is absence of the right atrioventricular connection (Orie et al., 1995). It follows, therefore, that the next key stage of normal development is the formation of a discrete inlet for the developing right ventricle, followed by formation of an outlet for the developing left ventricle. This requires significant remodeling of the initial interventricular communication (Anderson et al., 2019).

## 5 REMODELING OF THE INTERVENTRICULAR FORAMEN

**FIGURE 7:**
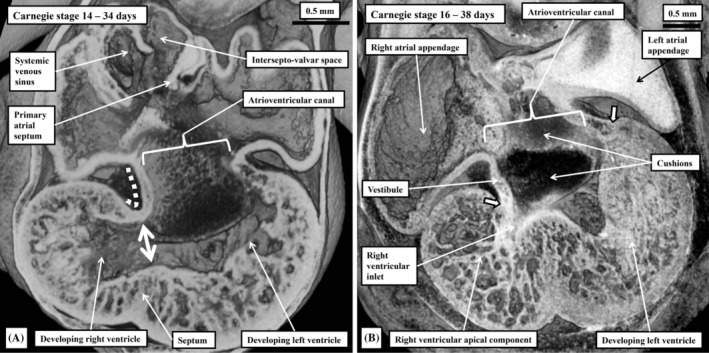
The images are four‐chamber sections taken through episcopic datasets prepared from human embryos at Carnegie stages 14 (panel A) and 16 (panel B). They show how expansion of the atrioventricular canal provides the developing right ventricle with its own inlet. This is because, at the stage when the primary interventricular foramen (double headed arrow in panel A) provides the outlet for the developing left ventricle, already the parietal walls of the right atrium and ventricle are in continuity (dashed white line in panel A). The images also show how, after expansion, the atrioventricular canal myocardium becomes sequestrated as the atrial vestibules subsequent to separation of myocardial continuity at the developing atrioventricular junctions (white arrows with black borders in panel B). The separation, however, is not completed until the fetal period of development (see Figure 15).

**FIGURE 8:**
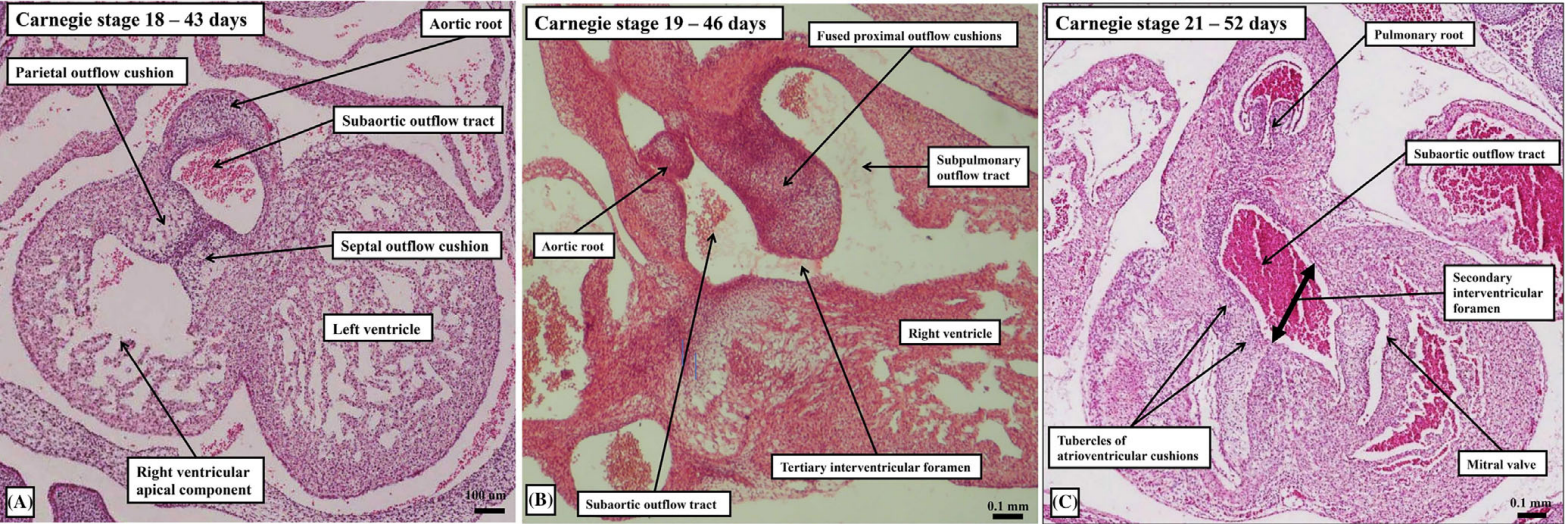
The histological sections, taken from embryos spanning the period between Carnegie stages 18 (panel A) and 21 (panel C), show how the embryo constructs a shelf in the roof of the right ventricle (panel A) so as to commit the subaortic outflow tract (panel B) to the developing left ventricle. Panels (A) and (C) are shown in the frontal projection, whereas panel (B) is prepared using the subcostal oblique orientation. As the shelf is formed within the cavity of the right ventricle, seen as a vertical structure in panel (B), a communication persists between the aortic root and the right ventricle. This is the tertiary interventricular communication. The space is closed by tubercles derived from the atrioventricular cushions, which form the membranous part of the septum (panel C). Once the communication is closed, the initial secondary interventricular communication (panel C) becomes the outflow tract for the definitive left ventricle.

The process of ballooning not only produces the ventricular apical components, but also the primordium of the muscular ventricular septum. It is the appearance of the septum that permits description of the primary interventricular foramen, which provides the inlet to the developing right ventricle (Figure 3B). The foramen has the crest of the ventricular septum as its caudal boundary, and the inner heart curvature as its roof (Figure 3A). It follows that, initially, all the blood entering the developing left ventricle must pass through this primary foramen so as to enter the developing right ventricle, which supports the entirety of the developing outflow tract (Figure 3B). The right ventricle achieves its own inlet by expansion of the atrioventricular canal (Lamers et al., 1992). From the outset of development, the parietal wall of the developing right ventricle, in the inner curvature, is in continuity with the parietal wall of the right atrium (Figure 7A). The expansion needed to provide the right ventricle with its own inlet occurs relatively early, during the period around Carnegie stage 14 (Figure 7B). As was shown in the study in which it proved possible to track the remodeling of the surrounds of the primary interventricular foramen on the basis of the reaction to an antibody to the GlN2 epitope (Lamers et al., 1992), the dorsal component of the myocardial boundary of the initial foramen became incorporated within the newly formed vestibule of the tricuspid valve. The remainder of the primary foramen can then be recognized at Carnegie stage 16 as the secondary foramen (Figure 7B). It continues at this stage to provide the outlet for the left ventricle, since the entirety of the outflow tract remains supported above the cavity of the developing right ventricle. So as to create an outlet for the developing left ventricle, the embryo follows the steps taken by the cardiac surgeon repairing the heart in the setting of double outlet right ventricle (Anderson, Lamers, et al., 2024). Muscularization of the fusing proximal outflow cushions (Figure 8A) produces a shelf in the roof of the right ventricle, in this way committing part of the ventricular cavity to the aortic root. A space remains, however, between the developing aortic root and the cavity of the apical part of the right ventricle (Figure 8B). This aorto‐right ventricular communication now represents a tertiary interventricular communication, since it is the ventral part of the initial channel between the ventricles. The aorto‐left ventricular communication at this stage, of course, remains as the secondary interventricular communication. It is closure of the aorto‐right ventricular communication, or the tertiary interventricular communication, by tubercles derived from the atrioventricular cushions (Figure 8C) that completes the process of ventricular septation (Odgers, 1938). The area formed from the tubercles will become the membranous part of the septum. With closure of the aorto‐right ventricular communication, which has been completed by Carnegie stage 21, at around 7 weeks after conception, the secondary interventricular foramen has become the outflow tract of the left ventricle (Figure 8C). The subaortic cavity, nonetheless, was initially part of the right ventricle (Figure 8A). Should the tubercles of the atrioventricular cushions fail to close the aorto‐right ventricular communication, the space persists as the perimembranous ventricular septal defect. It is then variability in the deficiencies of the muscular septum bordering the space that determines whether the defect opens centrally or is committed primarily to the right ventricular inlet or outlet (Tretter et al., 2019). If the muscular septal deficiency is extreme, the defect can be confluent, opening into all parts of the right ventricular cavity. As we will describe further when we return to development of the outflow tract, with the completion of ventricular septation, the surface, or envelope, of the muscularized proximal cushions is converted into the free‐standing subpulmonary infundibulum. This process also involves the conversion, presumably by apoptosis, of the core of the fused cushion mass into the fibro‐fatty extracavitary tissues that eventually separate the infundibular sleeve from the aortic root. Should septation be incomplete, however, the proximal cushions are able to form the muscular outlet septum. Should they fail to muscularise, the tissues can persist as a fibrous outlet septum. This is the arrangement seen when ventricular septal defects are juxta‐arterial (Tretter & Anderson, 2018).

## 6 ATRIAL AND ATRIOVENTRICULAR SEPTATION

**FIGURE 9:**
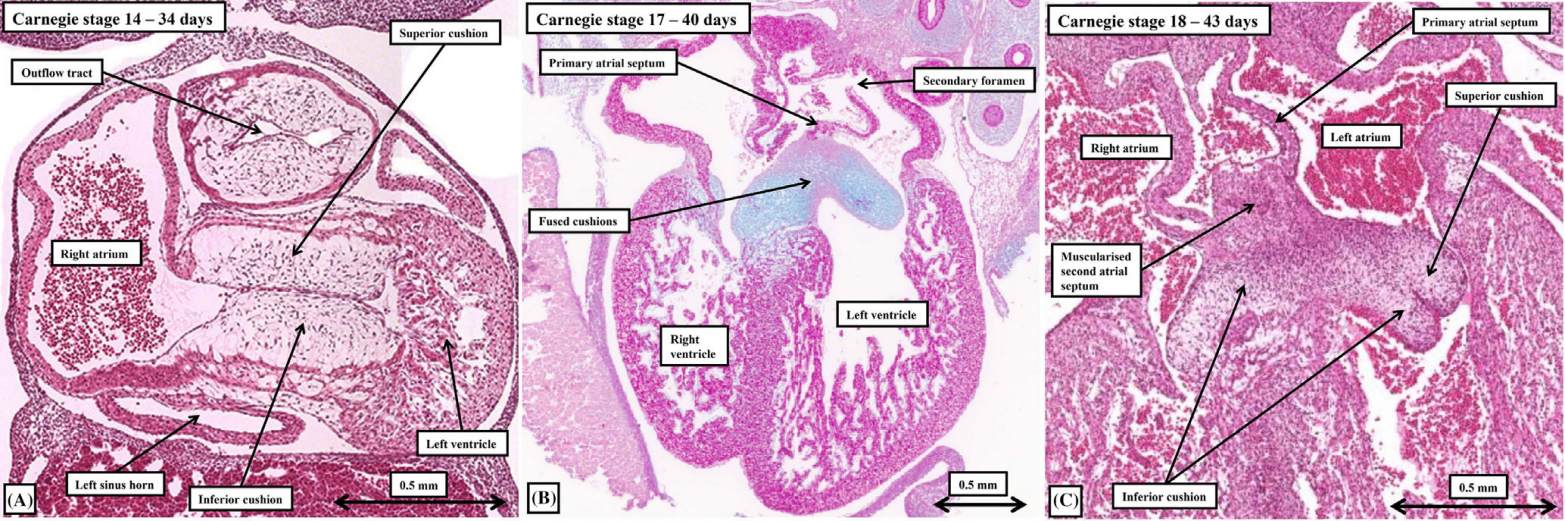
The histological sections, taken from embryos spanning the periods from Carnegie stage 14 (panel A) and 18 (panel C) show how the major atrioventricular cushions fuse to provide a landing zone for the developing atrial septum. Panel (A) is cut in frontal fashion to show the cushions lying edge‐to‐edge in supero‐inferior fashion within the atrioventricular canal at the end of the fifth week of development. By the end of the 6th week, the cushions have fused, as shown in panel (B), cut in the four‐chamber plane, and stained using the Periodic acid‐Schiff technique. The larger parts of the fused cushions overlie the cavity of the left ventricle, with their midpoint providing the landing zone for the leading edge of the primary atrial septum. As shown in panel (C), again cut in the four chamber plane, the mesenchymal cap and vestibular spine, which reinforce the leading edge of the primary septum (see Figure 10), muscularize to form the second atrial septum. Panel (C) also shows how the cushions have fused in the left ventricle to produce the aortic leaflet of the mitral valve, with the notch showing the final site of fusion.

**FIGURE 10:**
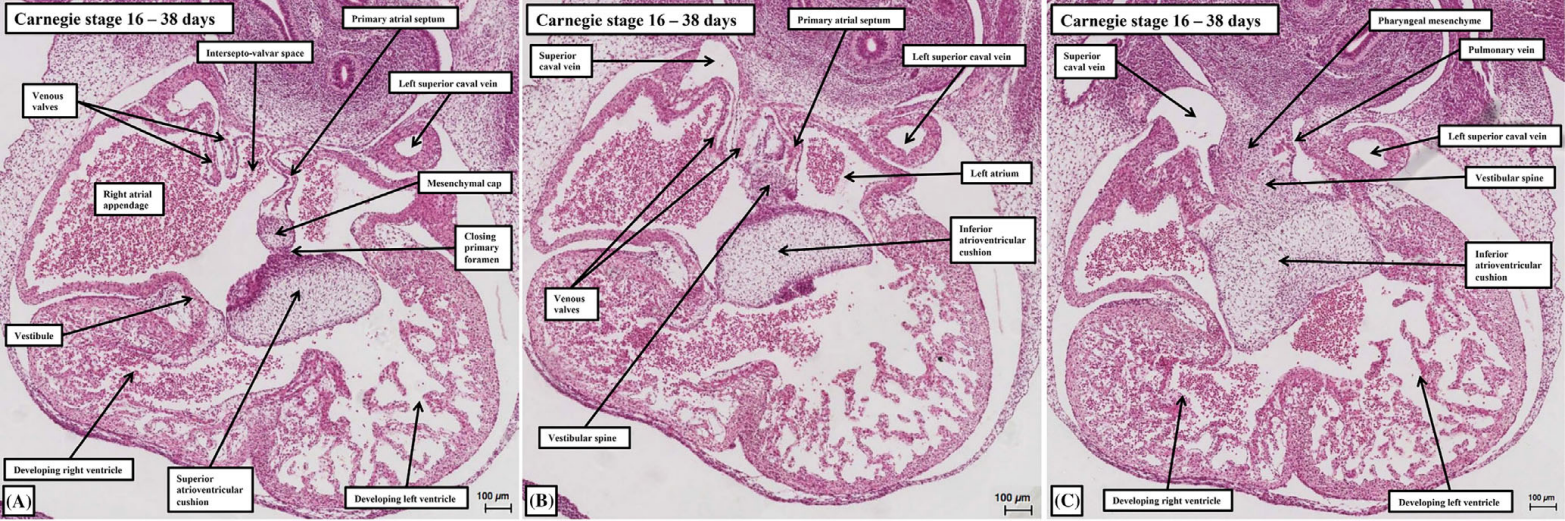
The serial sections are all from the same dataset, prepared from a human embryo at Carnegie stage 16. The sections pass from ventral (panel A) to dorsal (panel C). They show the mesenchymal cap on the leading edge of the primary atrial septum closing the primary foramen (panel A), with growth of the vestibular spine into the cavity of the right atrium along the right side of the primary septum (panel B). Panel (C) shows the continuity between the spine, the inferior cushion, and the pharyngeal mesenchyme, with the fusion of these components committing the pulmonary vein to the developing left atrium. Note that the left superior caval vein has its own walls, separate from the walls of the left atrium.

**FIGURE 11:**
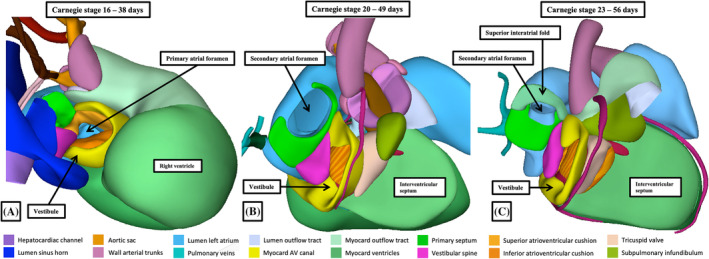
The reconstructions are made from the interactive pdf files prepared for the embryos at Carnegie stages 16 (panel A), 20 (panel B), and 23 (panel C). They show the steps involved in production of the definitive atrial septum. Panel (A) shows the primary atrial foramen, bounded by the mesenchymal cap and the atrioventricular cushions, with the vestibular spine growing into the right atrial cavity. Panel (B) shows how the spine reinforces the right side of the septum subsequent to closure of the primary atrial foramen, with the cranial margin of the primary septum breaking down to provide the secondary foramen. Panel (C) shows how it is the migration of the pulmonary veins to the roof of the left atrium that produces the superior interatrial fold, often mistaken for the second atrial septum. It is the muscularized spine and cap that produce the true second atrial septum (see Figures 9 and 10).

We described in the previous section how remodeling of the boundaries of the primary interventricular foramen set the scene for the completion of ventricular septation. An integral part of that septation was the fusion of the proximal outflow cushions, creating the shelf in the roof of the right ventricle which committed the subaortic area to the left ventricle. This represents septation from the arterial pole toward the interventricular communication. There is a similar process of septation at the venous pole, which produces septation of the atrial chambers subsequent to separation of the atrioventricular canal into the right and left atrioventricular junctions. The initial stage for septation of the venous components, however, is set by the remodeling of the systemic venous tributaries such that they open exclusively into the right side of the primary atrial component of the initial heart tube (Figure 5). Once the systemic venous return is committed to the developing right atrium, which has been achieved by Carnegie stage 15, the primary atrial septum is able to grow from the roof of the primary atrial component of the heart tube. As it grows toward the atrioventricular canal, it carries on its leading edge a mesenchymal cap. Its course takes it between the boundary of the systemic venous sinus, marked by the venous valves (Figure 5), and the opening of the pulmonary vein between the margins of the dorsal mesocardium (Figures 3 and 6). During these processes, the atrioventricular canal has been separated into the right and left atrioventricular orifices by the formation of cushions in its lumen. Initially, the two cushions lie edge‐to‐edge in superior to inferior fashion (Figure 9A). By Carnegie stage 17, when almost 6 weeks have elapsed subsequent to conception, the cushions have fused, creating a landing zone for the leading edge of the primary atrial septum (Figure 9B). By the beginning of the seventh week of development, at Carnegie stage 18, the mesenchymal tissues reinforcing the leading edge of the septum have muscularized, creating a second atrial septum (Figure 9C). There are two components to this second atrial septum. The first is the mesenchymal cap carried on its leading edge, which from the outset is in continuity with the superior atrioventricular cushions (Figure 10A). The second component enters the heart through the right margin of the dorsal mesocardium. It grows into the atrial cavity as a vestibular spine (Figure 10B). It was described in this fashion by His as along ago as the latter part of the 19th century (Kim et al., 2001), but is now frequently labeled as the dorsal mesenchymal protrusion (Snarr et al., 2007) Reinforcing the attachment between the mesenchymal cap and the superior cushion, it lies along the inferior cushion, committing the orifice of the pulmonary vein to the developing left atrium (Figure 10C). When first growing from the atrial roof, the space between the mesenchymal cap on the leading edge of the primary septum and the atrioventricular cushions is the primary atrial foramen, or the “ostium primum” (Figure 11A). So as to continue to provide a route for the richly oxygenated placental flow to reach the left side, the cranial part of the primary septum breaks away from the atrial roof, thus producing the secondary atrial foramen (Figures 9B and 11B). Not until the end of the embryonic period, however, at Carnegie stage 23, does a fold appear between the atrial chambers to provide a buttress against which the superior edge of the primary septum can abut in postnatal life to close the oval foramen. This fold is often described as the second atrial septum. It is muscularization of the mesenchymal cap and the vestibular spine that produce the true second atrial septum (Jensen et al., 2017). This structure forms the prominent antero‐inferior buttress of the oval fossa (Figure 11C). The formation of the superior margin of the fossa, which is a fold rather than a septum, depends on the appropriate migration of the pulmonary veins to the atrial roof concomitant with the “cannibalization” of the right and left veins (Figure 11B,C).

## 7 REMODELING OF THE VENTRICULAR OUTFLOW TRACT

**FIGURE 12:**
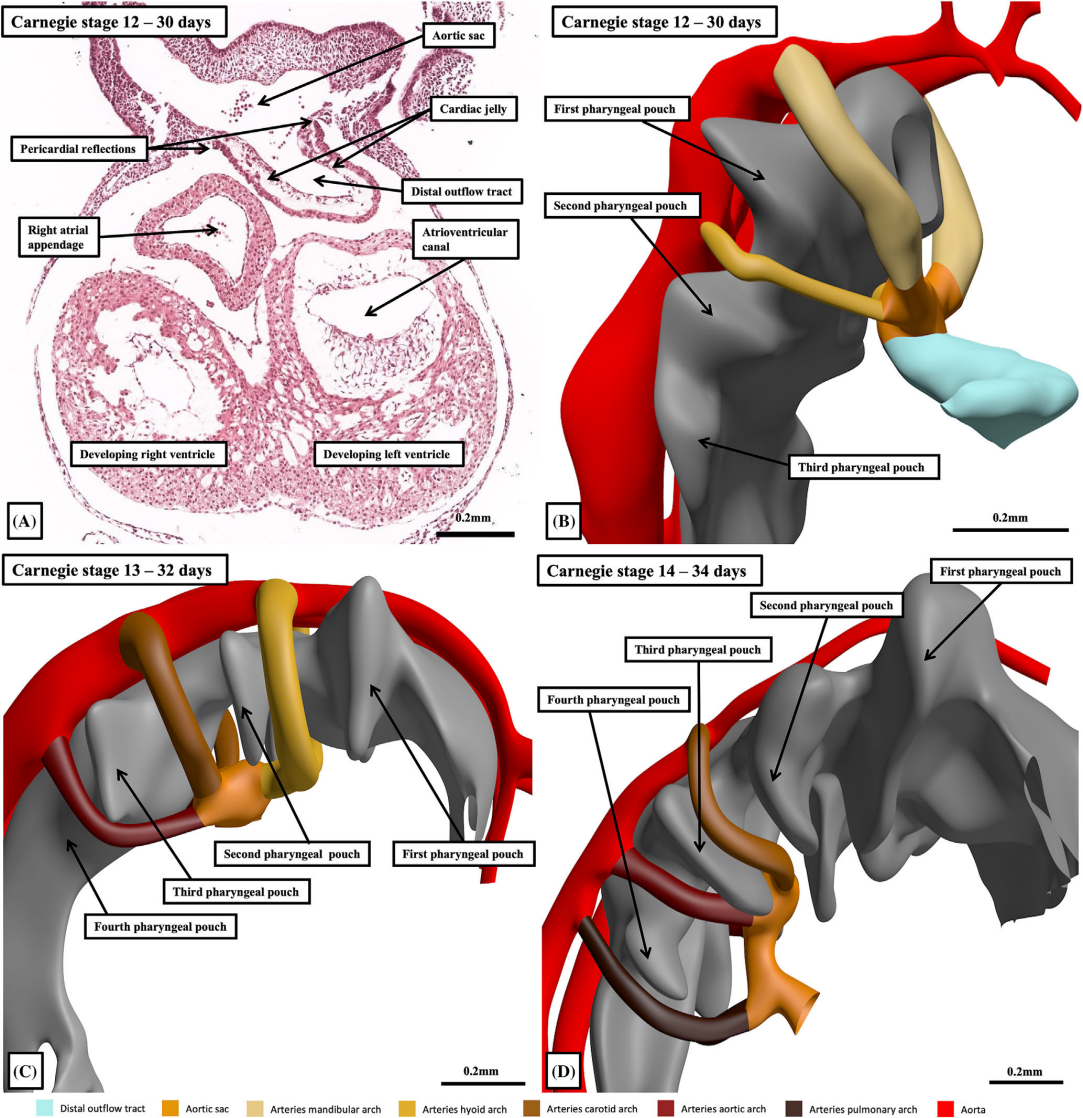
The histological section in the frontal plane (panel A) and the reconstructions of the interactive pdf files (panels B–D) show the sequence of development of the arteries of the pharyngeal arches. Panel A shows the union initially between the myocardial distal outflow tract and the aortic sac. Panels (B)–(D) show that there are only five pharyngeal arches formed during normal development. Because of the confusion caused by the initial acceptance of the concept of six sets of arteries, it is better to name the arteries as shown rather than to number them.

As we have already described, at Carnegie stage 12, at the beginning of the fifth week of development, the outflow tract extends as part of the primary heart tube, with a solitary lumen, to the margins of the pericardial cavity, where its cavity is continuous with the cavity of the aortic sac. The walls of the outflow tract at this stage are exclusively myocardial. A layer of cardiac jelly can be seen encircling the lumen (Figure 12A). The aortic sac itself, at this stage, gives rise to two sets of pharyngeal arch arteries. The arches themselves are blocks of mesoderm, also containing mesenchyme derived from the neural crest, which are flanked by pharyngeal pouches (Graham et al., 2019), with the first arch formed cranial to the first pouch (Figure 12B). This first arch is mandibular. The second pair passes through the hyoid arches (Figure 12B). Already by the next stage of development, after around 32 days, the arteries within the mandibular arches have virtually disappeared. By now, however, two new sets of arteries have been formed within the developing carotid and aortic pharyngeal arches (Figure 12C). With the advent of Carnegie stage 14, after about 34 days, only the dorsal part of the arteries of the second arches remains. A final set of arteries has now formed within the ultimate, or pulmonary, pharyngeal arches (Figure 12D). It is customary to describe this final pair as the arteries of the sixth arches. At no stage of development, however, is there any formation of a fifth pharyngeal pouch (Anderson et al., 2023). Only four discrete arches can be seen, with the ultimate pair of arteries percolating through the mesenchyme forming the pulmonary arch caudal to the fourth pouch. By Carnegie stage 15, at the beginning of the sixth week of development, the arteries of the pulmonary arches have given rise to the right and left pulmonary arteries, which extend through the ventral pharyngeal mesenchyme to join the developing lungs. It is the arteries of the caudal three sets of arches, which can be described as being carotid, aortic, and pulmonary, that remodel to form the definitive extrapericardial systemic and pulmonary pathways. By this stage, the arteries initially present in the mandibular and hyoid arches have effectively disappeared. There is never any formation of a fifth pharyngeal arch within a series of six arches, as is usually shown in the so‐called Rathke diagram. In fact, Rathke himself showed only five sets of arteries when producing his concept (Anderson et al., 2023). It is a mistake, therefore, to described abnormal extrapericardial pathways as “persistent fifth arch arteries.” It is the left‐sided artery of the true fifth pharyngeal arch that persists as the arterial duct. Because of the frequent description of alleged fifth arch arteries, it would now be confusing to seek to rename that duct as the “fifth arch artery,” even though this would be developmentally correct. For this reason, it is better now to name the developing arch arteries, as above, rather than numbering them (Graham et al., 2023).

During the stages from Carnegie 12 through 14, all of the bilaterally symmetrical arch arteries terminate in the dorsal aortas, which are themselves bilateral structures, although they merge caudally. By stage 13, they have fused caudally to the level of the umbilical arteries, at the 4th lumbar segment. Intersegmental arteries are now recognizable, with the seventh cervical arteries, by stage 14, becoming larger than their neighbors. These vessels will eventually become the subclavian arteries. By Carnegie stage 16, the arteries have begun to lose their symmetry, with loss of the right‐sided channels. The right dorsal aorta distal to the 7th segmental artery has disappeared at Carnegie stage 20, equivalent to 49 days of development, with the right pulmonary arch artery also disappearing at around this stage. The aortic sac has itself, by Carnegie stage 20, undergone significant remodeling. Its cranial component has developed horns, with the right horn becoming the brachiocephalic artery, and the left horn the transverse aortic arch. Over the same period of time, the left seventh segmental artery, which becomes the left subclavian artery, crosses the junction of the artery of the left pulmonary arch with the descending aorta. The left pulmonary arch artery itself persists as the arterial duct, usually becoming ligamentous postnatally. At 7 weeks of development, therefore, the major arterial vessels have adopted their definitive configuration.

**FIGURE 13:**
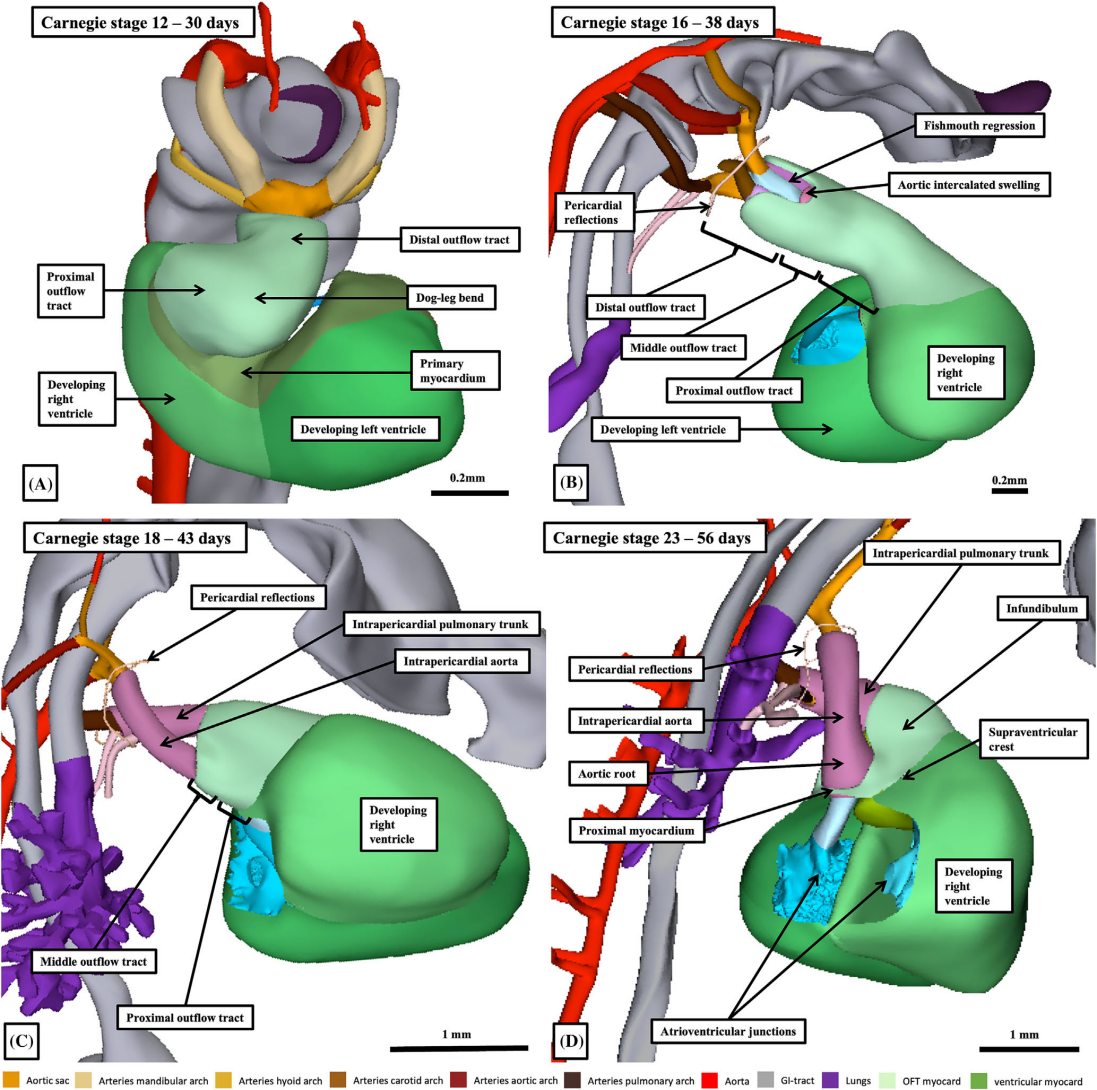
The reconstructions made from the interactive pdf files show the effective regression of the myocardial border relative to the pericardial reflections. At Carnegie stage 12 (panel A), the myocardial walls extend to the margins of the aortic sac. By Carnegie stage 16, there has been further contributions of cells to the outflow tract from the second heart field, but producing non‐myocardial tissues which fill the jaws of the fishmouth configuration of the distal myocardial border. This permits recognition of a tripartite outflow tract. As shown in panel (C), the arterial roots form in the middle part of the outflow tract, which has retained its myocardial walls. By Carnegie stage 23 (panel D), the myocardial walls form mainly the infundibulum of the right ventricle, although still at this stage the aortic root has retained a short infundibulum.

**FIGURE 14:**
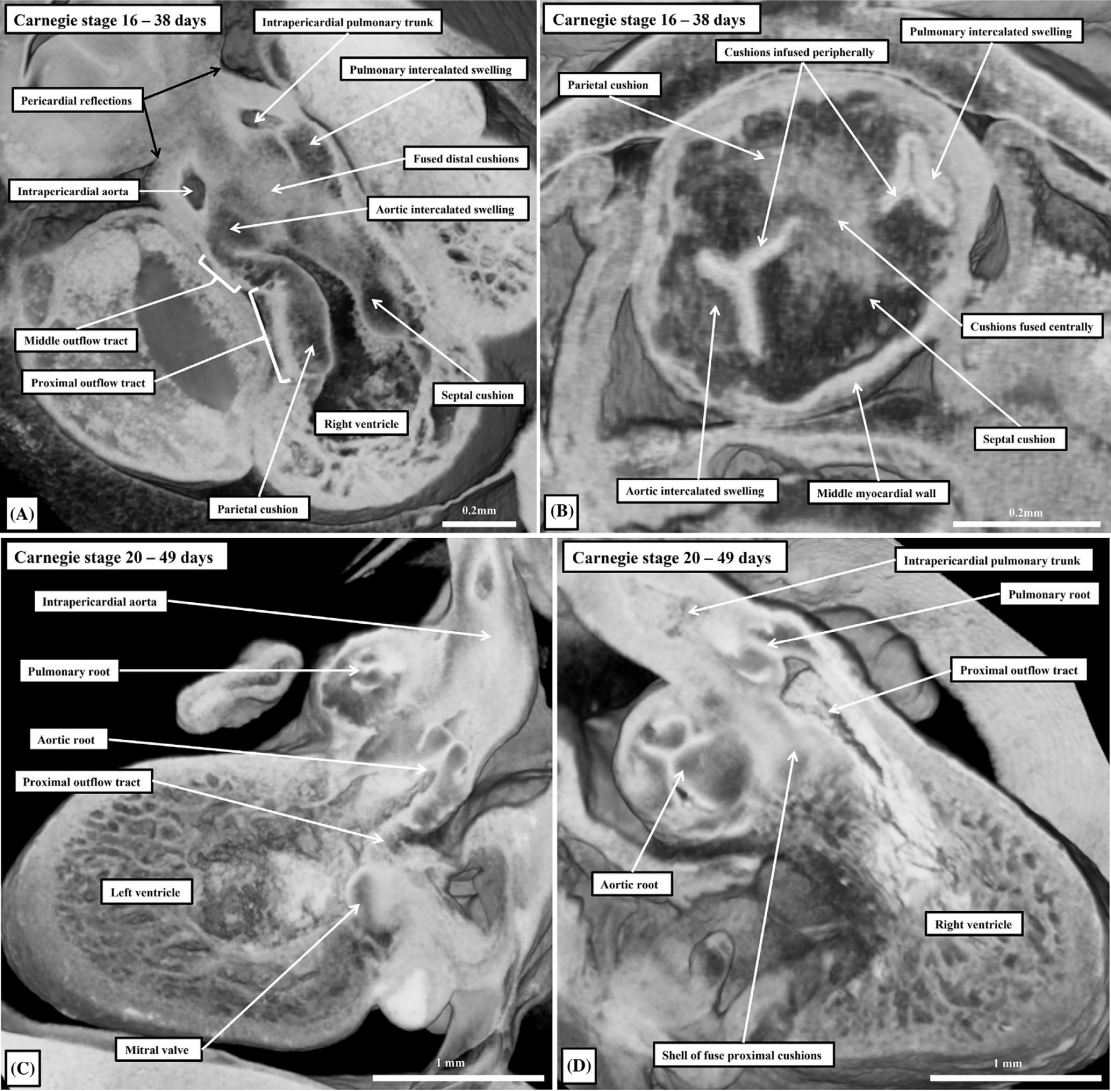
The sections are taken from episcopic datasets prepared from human embryos at Carnegie stages 16 (panels A and B) and 20 (panels C and D). Panels (C) and (D) are from the same embryo. The embryos in panels (A) and (B) are more mature from the one reconstructed and shown in Figure 13. The images show the tripartite outflow tract (panel A), the formation of the arterial roots as seen in short axis (panel B), and the developing leaflets of the arterial valves (panels C and D—both in long axis).

While all these changes have been taking place extrapericardially, there has also been major remodeling of the intrapericardial outflow tract. Coincident with the appearance of the first pharyngeal pouch at Carnegie stage 11, new cells enter the arterial pole of the heart from the second heart field (Meilhac et al., 2004). This produces a fourfold increase in its length over the period of 8 days between Carnegie stages 12 and 16. With this marked growth, there is a disappearance of the initially obvious dog‐leg bend between the proximal and distal components of the myocardial outflow tract, with the outflow tract straightening between Carnegie stages 12 and 15. The majority of the new material added to the outflow tract, however, is non‐myocardial. It will form the arterial components of the outflow tracts. As we initially emphasized, with the appearance of the distal non‐myocardial component, it becomes possible to recognize three parts of the outflow tract (Figure 13A). Between Carnegie stages 15 and 20, there is then minimal growth of the persisting middle and proximal myocardial components. The overall architecture, nonetheless, changes markedly. Within 2 days, between Carnegie stages 16 and 17, the overall tube changes from being narrow, with a solitary lumen, to widening into a figure‐of‐eight configuration when its distal non‐myocardial part is seen in short axis. The waist in the figure‐of‐eight then represents the developing separation between the intrapericardial aortic and pulmonary trunks. The decrease in length occurs mostly in its proximal portion. Concomitant with the change, there is also a marked proximal shift in the distal boundary of the myocardial part relative to the margins of the pericardial cavity (Figure 14).

Up to, and including, Carnegie stage 13 the distal myocardial boundary reaches to the pericardial reflection (Figure 14A). The initial regression of the boundary reflects the appearance of non‐myocardial parietal tongues. These appear initially as cranial and caudal spurs, but as they increase in size, they shift to form the non‐myocardial right and left walls of the distal outflow tract, interdigitating with the persisting myocardial walls in fishmouth fashion (Figure 14B). At the same time as the tongues produce the parietal walls, so a protrusion from the dorsal wall of the aortic sac grows into the cavity of the distal outflow tract as an aortopulmonary septum, separating the cranial and caudal components of the aortic sac itself. When first observed, at Carnegie stage 14, the protrusion has a transverse orientation. As it grows into the distal outflow tract, its orientation gradually changes in anti‐clockwise direction when seen from the ventral aspect, placing the aortic and pulmonary components of the aortic sac into rightward and cranial and leftward and caudal positions by stage 16. With further growth, these have become right and left sided at Carnegie stage 17 (Figure 13A). Already by Carnegie stage 16, the aortopulmonary septum has fused with the distal ends of the cushions, which themselves have been cellularized by cells derived from the neural crest and the endocardium, albeit contained within the part of the outflow tract that retained its myocardial walls. The cushions themselves, when first formed, spiral throughout the myocardial outflow tract, with the inferior cushion distally achieving a septal location within the right ventricle. By the time that the aortopulmonary septum has fused with the distal ends of the cushions, so have the cushions themselves fused distally (Figure 13B). And, by this time, additional swellings have grown from the distal ends of the non‐myocardial parietal tongues, with the swellings interdigitating in the middle part of the outflow tract to produce the primordiums of the arterial roots (Figure 13B). The intercalated swellings remain relatively small in terms of their length during stages 14 and 15, but increase in size from stages 16 onwards as they begin their transformation into the non‐adjacent leaflets of the developing arterial roots. The distal parts of the major outflow cushions undergo comparable transformation to produce the adjacent leaflets of the valves (Figure 13C,D). Further proliferation of the non‐myocardial walls then produced the valvar sinuses within the middle part of the outflow tract, with this, in turn, leading to still further proximal regression of the distal myocardial border (Figure 14D).

Fusion of the cushions within the proximal part of the myocardial outflow tract takes place during Carnegie stages 16 through 18. As we have already described, it is this fusion of the proximal cushions that produces the shelf in the roof of the right ventricle, thus committing the subaortic area as the left ventricular outflow tract (Figure 8). During this process, there is muscularization of both the proximal and distal parts of the cushions. The muscularized proximal cushions become the free‐standing subpulmonary infundibulum. The muscularized distal cushions provide the support for the pulmonary root, as it is separated from the aortic root, and for the aortic root as it is committed to the left ventricle. The newly‐formed myocardium on the aortic side is then incorporated into the crest of the muscular ventricular septum. Again, as we have already stressed, if development proceeds normally, the embryonic outlet septum transforms into the supraventricular crest and the free‐standing muscular infundibulum. With these changes, the embryonic outlet septum, formed initially by the fused outflow cushions, can no longer be considered a septum. Instead, it has largely been incorporated into the right ventricle as the infundibulum.

## 8 FURTHER CHANGES DURING THE PERIOD OF EARLY FETAL DEVELOPMENT

**FIGURE 15:**
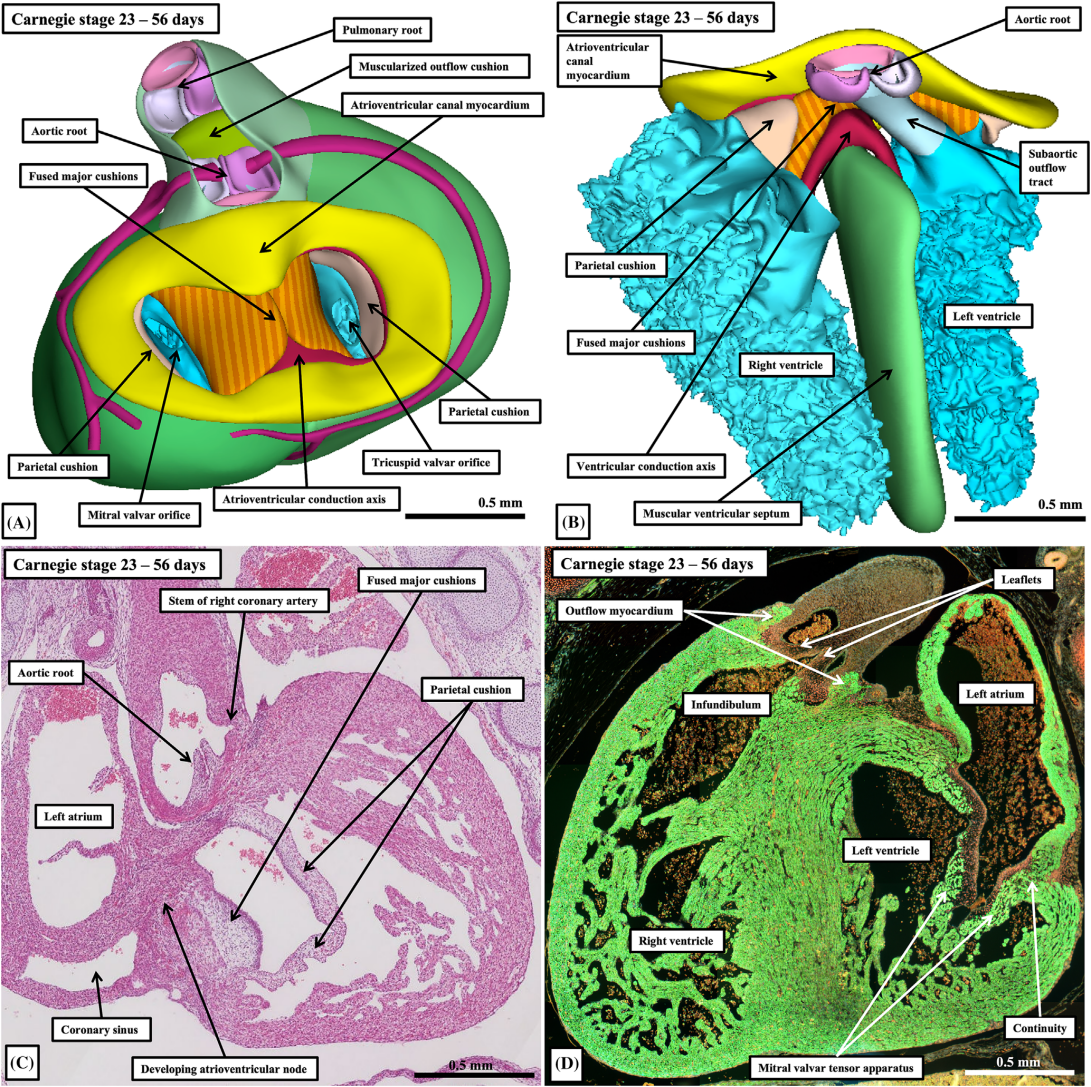
The images are all from human embryos at Carnegie stage 23, which is at the end of the embryonic period of development. Panels (A) and (B) are from the interactive pdf file. They show that, although septation is complete by the end of the embryonic period, the atrioventricular canal has yet to expand inferior to produce the inferior pyramidal space, with the conduction axis emerging at the crux as viewed from above looking toward the ventricular apex (panel A). As can be seen in both panels (A) and (B), with panel (B) showing the aortic root relative to the atrioventricular canal as viewed from the front, the root has yet to be “wedged” between the developing mitral valve and the ventricular septum. Panels (C) and (D) are histological sections from the same dataset, with panel (C) stained with hematoxylin and eosin, but panel (D) processed to show myocardium in green. They are oriented as seen from the right side (panel C) and the left side (panel D). At this stage, there has been no delamination of the septal leaflet of the tricuspid valve (panel C), while the developing tension apparatus of the mitral valve remains largely myocardial, with myocardial continuity still present across the developing left atrioventricular junction. The section also shows the outflow myocardium still supporting the developing leaflets of the pulmonary valve.

It might be thought that, with the completion of septation, during the period from Carnegie stages 20 through 23, the heart would be in its definitive state by the end of the embryonic period. This is far from the case. It is certainly possible to recognize, at this stage, the key morphological features of the atrial chambers, namely the extent of the pectinate muscles relative to the atrioventricular junctions. The atrioventricular junctions themselves, however, are not expanded to the extent found in the postnatal heart. Rather, the arrangement at the end of the embryonic period, when the inferior buttress of the atrial septum is firmly attached to the landing zone provided by the fused atrioventricular cushions (Figure 11C), is reminiscent of the common atrioventricular junction found as the phenotypic feature of deficient atrioventricular septation (Figure 15A; Anderson et al., 2010). At the end of the embryonic period, there has been no formation of the inferior pyramidal space (Tretter et al., 2022). And the aortic root, as in atrioventricular septal defect with common atrioventricular junction, has yet to be wedged in the left ventricle between the leaflets of the developing mitral valve and the ventricular septum (Figure 15B). The major difference between the normal and abnormal hearts, of course, is that septation is complete, and the left atrioventricular valve is already bifoliate (Figure 15A), rather than resembling the trifoliate pattern which is another of the phenotypic features of deficient atrioventricular septation (Anderson et al., 2010). During the fetal period of development, there must be ongoing inferior expansion of the atrioventricular junctions, and ongoing attenuation of the outflow tract myocardium in the roof of the left ventricle to produce the aortic‐to‐mitral valvar continuity, which is one of the features of the definitive left ventricle (Crucean et al., 2024). Already at the end of the embryonic period it is possible to recognize the distinction in the trabecular pattern of the developing right and left ventricles, but the extent of the trabecular component is greater at this stage of development than at the end of gestation (Figure 15D). The tension apparatus of the atrioventricular valves is still relatively immature, with no delamination of the septal leaflet of the tricuspid valve (Figure 15C). The eventual arrangement also indicates that the postnatal trifoliate pattern of the tricuspid valve depends on the ongoing expansion of the atrioventricular junction, rather than the valve possessing three primordiums (Figure 15A). There are many other changes requiring significant remodeling during the early period of fetal development. Thus, at the end of the embryonic period, the stems of the major right and left coronary arteries have yet to be fully incorporated within the developing sinuses of the aortic root (Figure 15C). And the developing atrioventricular node is positioned at the crux of the heart (Figure 15A,C), rather than being centrally located as seen postnatally. Much work remains to be done, therefore, to explain the remodeling required during fetal life to produce the postnatal arrangement.

## 9 DISCUSSION

As long ago as the first part of the 20th century, Maude Abbott pointed to the value of knowledge of cardiac development in providing the basis for understanding the abnormal findings encountered when the heart is congenitally malformed (Abbott, 1934). In an inflammatory book chapter published in the latter decades of the 20th century, one of us argued that embryology was, in fact, a hindrance rather than a help in providing such understanding (Becker & Anderson, 1984). At the time, there was some justification for this stance, since many of the embryological concepts were based on armchair speculation, rather than on new evidence emerging from the workbench. And, as we emphasized in our introduction, it is not easy to unravel the remarkable temporal and three‐dimensional changes seen during cardiac development simply on the analysis of serial histological sections. All this has changed with the turn of the centuries. As we now demonstrate, it is possible to take the developing heart apart, and put it back together again, using the interactive pdf files that we produced to supplement our earlier pictorial account of human development (Hikspoors et al., 2022). The serially sectioned human embryos are themselves now available for general inspection in the website of the Human Developmental Biology Resource (Anderson, Kerwin, et al., 2024). And, it is becoming possible to identify the presence of key genes in the human datasets (Sizarov et al., 2010; Wessels et al., 1990), which permit comparisons to be made with the multitude of investigations that have been published using normal and genetically modified mouse embryos. Taken together, as hopefully, we have shown in our review, this now permits deductions to be made relative to the majority, if not all, the various congenital cardiac malformations. For example, the initial development of the apical trabecular component of the right ventricle now provides the evidence to show that the small chamber found in functionally univentricular hearts when the left ventricle is dominant is an incomplete right ventricle. The stages described for transfer of the aortic root then provide the evidence required to understand the nature of the channels between the ventricles as seen in the settings of tetralogy of Fallot and double outlet right ventricle. As we have also stressed, however, the normal arrangement is not reached at the embryonic period of development. More work is needed to elucidate the changes that occur during the period of early fetal development, with these changes potentially contributing to a greater understanding of the morphogenesis of lesions such as Ebstein's malformation.
